# Curricular Considerations: The Process of Integrating Simulation-Based Learning Into a Social Work Communication and Interviewing Skills Course

**DOI:** 10.7759/cureus.19191

**Published:** 2021-11-01

**Authors:** Michelle Skop, Eva Peisachovich, Liming Cao

**Affiliations:** 1 Faculty of Social Work, Wilfrid Laurier University, Brantford, CAN; 2 Medical Education and Simulation, York University, Toronto, CAN

**Keywords:** communication skills, course design, pedagogy, active learning, standardized person methodology, interdisciplinary education, social work education, simulation-based learning

## Abstract

Simulation-based learning (SBL) is used as an educational tool within health professions education disciplines, including medicine and nursing. More recently, SBL has been applied within social work education as a growing body of research, demonstrating its efficacy in teaching social work competencies. SBL provides students with safe and practical opportunities to apply their skills within highly realistic settings. The growing body of literature on SBL within social work education informed the development of a new Bachelor of Social Work (BSW) course focused on communication and interviewing skills at Wilfrid Laurier University. The purpose of this editorial is to provide an example of a collaborative process for integrating simulation as a pedagogy within course design. This collaborative process involved four stages: designing the course, preparing, and revising the simulations, facilitating the simulations, and evaluating student learning and experience. This editorial may assist instructors by providing a pedagogical framework for incorporating SBL into both new and existing curricula.

## Editorial

Background

Simulation-based learning (SBL) is used as an educational tool within health professions education disciplines, including medicine and nursing. More recently, SBL has been applied within social work education as a growing body of research, demonstrating its efficacy in teaching social work competencies [1]. The purpose of SBL is to provide students with safe and practical opportunities to apply their skills within highly realistic settings [2]. SBL can be integrated into a curriculum using role-play between students, computer-based simulations, and interviews with simulated persons (SPs) [2]. Interviews with SPs provide a more realistic experience for students to practice skills compared to role-playing with peers [3].

The growing body of literature on SBL within social work education informed the development of a new Bachelor of Social Work (BSW) course focused on communication and interviewing skills. The purpose of this editorial is to provide an example of a process of integrating simulation as a pedagogy within course design. This may assist instructors by providing a framework for incorporating SBL into both new and existing curricula.

Contextualizing social work education

Social work programs are uniquely situated to provide professional training grounded by the values and principles of social justice, equity, and anti-oppressive practices [4]. Social work critically explores the experiences and realities of individuals, families, groups, and communities within their historical, social, political, cultural, and economic contexts [4]. The BSW program at Wilfrid Laurier University is an accredited program located in Brantford, Ontario. This program prepares students for generalist social work practice in diverse settings, including health, mental health, schools, child welfare, and domestic violence prevention. The BSW program involves a progression from foundational knowledge about social welfare and social work ethics (year one) to micro- and macro-level theories and social policy (year two) to practice courses (year three) and social work elective courses (year four). Students also participate in two 12-week placements in the third and fourth years. BSW faculty and staff recognized that students required further practice opportunities in preparation for their third-year courses and placements. To address this need, strategies for embedding SBL into the curriculum were explored. This process involved: organizing a BSW working group, surveying BSW field supervisors about what key communication skills students needed to be successful in their placements, and consultation with social work colleagues using SBL at several Ontario universities. After these consultations, a communication and interviewing skills course was developed, and a research team was formed to co-design, implement, and evaluate role-play simulations embedded in the course. This social work project, which incorporated SBL as a pedagogy in a new course focused on communication and interviewing skills, became part of a larger interdisciplinary research study. The study used a mixed-methods research design to explore the impact of simulation on student learning and to refine future iterations of the course simulations. Since the focus of this editorial is simulation design and delivery, the research project will be discussed in future publications.

Embedding simulations into social work course design

During winter 2021, two sections (section one = 13 students and section two = 22 students) of the course were taught remotely using a synchronous approach due to COVID-19. The course was offered as an elective but will become a required course for all second-year BSW students beginning winter 2022. Weekly course topics included critical reflexivity, active listening skills (e.g., attending behaviours, paraphrasing/summarizing, asking questions, empathy, and use of silence), intercultural communication, holistic assessment, documentation, and virtual counselling skills. Each three-hour class involved an interactive lecture, discussion, and small group activities. During the first month, the concept of simulation was introduced, and students’ confidence to participate in the simulations was slowly developed and encouraged through role-plays and other active learning strategies. In these early weeks, students received an overview of what to expect during the simulations, which would occur halfway through the 12-week semester; this included a discussion of the case scenario, the objectives of the simulation, the process of observation, and the way feedback would be provided. Students were also invited to volunteer in the simulations.

Several weeks before each simulation, the project team met to practice and revise the case scenario. The scenario, in which a young adult experiencing interpersonal issues sought online social work support, was adapted to reflect issues exacerbated by the global pandemic, as well as the current realities of virtual service delivery. In the first simulation, students were tasked with building rapport and beginning a first interview, including discussing consent for social work service and confidentiality. Building off the same case scenario, in the second simulation, students were tasked with conducting a social work assessment by gathering information about the client's history while demonstrating active listening skills.

In weeks five and eight, the simulation classes began with music and an icebreaker activity to increase comfort and connection. The case scenario and objectives were reviewed, and all observing students were instructed to turn off their cameras and mute their microphones. During the simulation, three student volunteers spent 10 minutes each role-playing a social work placement student. When one student completed their 10-minute role-playing session, the next student continued where their peer left off. The volunteer students were invited to take time-outs as needed during their 10-minute sessions. They also received encouragement from their observing peers, who wrote messages of support using the Zoom chat function.

After the three students completed their role-play sessions, the team utilized the advocacy inquiry model (AIM) to facilitate a four-layered process of debriefing [5]. First, each of the three students shared their experience in the simulation with a focus on identifying one positive aspect of the interview and one area for improvement. Second, the SP provided feedback focused on communication skills (e.g., “when you did X, I felt X”). Third, the course instructor provided feedback on social work specific skills focused on one positive and one area for growth. At times, the feedback was synthesized when common themes were observed. Finally, the rest of the class was invited to share their reflections and observations. 

To promote integrated learning, each simulation was connected to an assignment. After the first simulation, students wrote a reflection paper identifying the communication skills they observed during the simulation. After the second simulation, students prepared a biopsychosocial-spiritual assessment based on the information learned across both simulations. To assist with assignment preparation, after the second simulation debrief, all students were invited to ask the SP questions to learn about aspects of their life, which were not addressed in the simulation. For the final assignment, students recorded themselves conducting a 10-minute social work interview with a peer; they also wrote a paper reflecting on the communication and interviewing skills they used, areas for improvement, and their growth over the semester.

During the final class, students were invited to provide feedback about their experiences of the simulations, as well as the course. This feedback will inform future iterations of the course.

**Figure 1:**
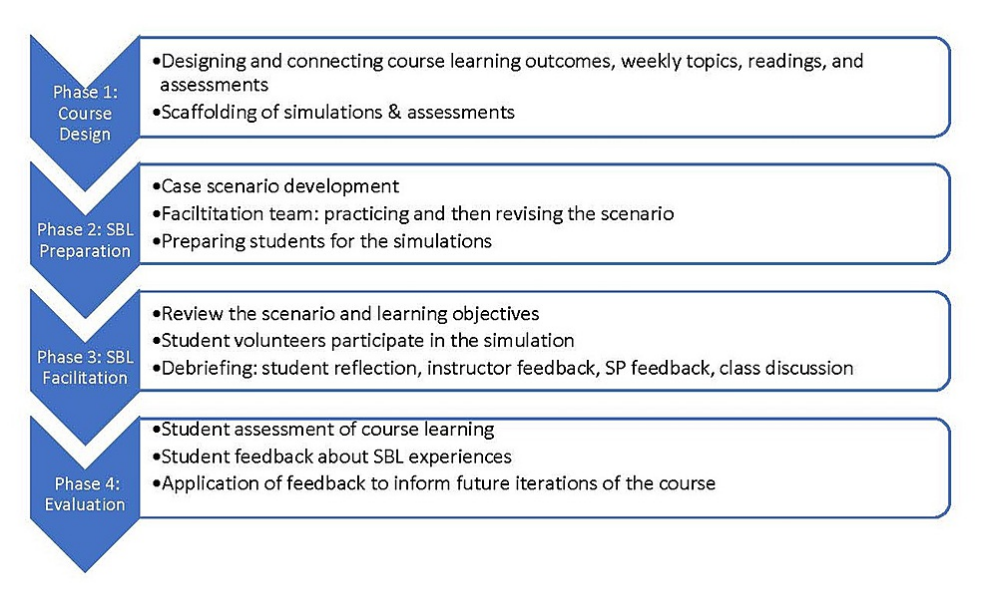
Phases of planning and implementing simulation-based learning

The process of designing the course, preparing and facilitating the simulations, as well as evaluating student learning and experience is represented in the following diagram (Figure 1).

Students as collaborators in course development and delivery

The course development and facilitation process involved students as collaborators. One of the team members completed her fourth year BSW placement as a Teaching Assistant in the course, and her role involved developing course content, co-facilitating classes, and sharing placement experiences to connect course content to real-life examples. During the 12-weeks of the course, the team observed students’ growth and increased confidence, as they engaged with the role-plays and simulations, as well as reflected on these experiences in their assignments. Before the simulations, students reported having limited practice experience, and thus they felt unprepared for and lacked confidence in their ability to begin a first interview, conduct an assessment, and build rapport with service users. The informal role plays combined with the two simulations provided students with clear illustrations of what interviewing could look like in practice. As a fellow student, the Teaching Assistant observed that this course met student needs by providing experiential learning opportunities to practice communication and interviewing skills early in the BSW program; this way students had time to reflect on their strengths and areas for improvement before their first-field placement.

Implications and future directions

This editorial described the process of integrating SBL into the social work curriculum as an exemplar for instructors, across a variety of disciplines, who may be considering the use of simulation in their courses. When deciding to integrate simulations, key considerations for course planning include ensuring adequate time to hire and train SPs, schedule the simulations, and develop and revise the case scenarios. Moreover, the authors recommend: (a) scaffolding the simulations within the course curriculum to slowly build student comfort and skills, and (b) connecting the simulations to course assignments to solidify course learning outcomes.

The simulations in this BSW course were designed to illustrate aspects of direct practice with individuals. Future directions for simulation design and research include exploring the application of simulations related to service delivery with families, groups, and communities. Another area for future exploration is students’ perceptions of how feedback is provided post-simulation, as well as the development of opportunities to apply this feedback.
